# Human Behavior, Livelihood, and Malaria Transmission in Two Sites of Papua New Guinea

**DOI:** 10.1093/infdis/jiaa402

**Published:** 2021-04-27

**Authors:** Daniela Rodríguez-Rodríguez, Michelle Katusele, Alma Auwun, Magdalene Marem, Leanne J Robinson, Moses Laman, Manuel W Hetzel, Justin Pulford

**Affiliations:** 1 Swiss Tropical and Public Health Institute, Basel, Switzerland; 2 University of Basel, Basel, Switzerland; 3 Papua New Guinea Institute of Medical Research, Goroka and Madang, Papua New Guinea; 4 Walter and Eliza Hall Institute of Medical Research, Melbourne, Australia; 5 Burnet Institute, Melbourne, Australia; 6 Liverpool School of Tropical Medicine, Liverpool, United Kingdom

**Keywords:** malaria, residual transmission, outdoor transmission, Papua New Guinea, human behavior, outdoor mosquito exposure, human-vector contact, LLINs

## Abstract

**Background:**

Malaria transmission is currently resurging in Papua New Guinea (PNG). In addition to intervention coverage, social and cultural factors influence changes in epidemiology of malaria in PNG. This study aimed to better understand the role of human behavior in relation to current malaria control efforts.

**Methods:**

A mixed-method design was used in 2 sites in PNG. In-depth interviews, focus group discussions, cross-sectional malaria indicator survey, and population census were implemented.

**Results:**

We identified 7 population groups based on demographics and behavioral patterns with potential relevance to Anopheles exposure. People spend a substantial amount of time outdoors or in semiopen structures. Between 4 pm and 8 am, all types of activities across all groups in both study sites may be exposing individuals to mosquito bites; sleeping under a long-lasting insecticidal net was the exception. The later in the night, the more outdoor presence was concentrated in adult men.

**Conclusions:**

Our findings highlight the potential of outdoor exposure to hamper malaria control as people spend a remarkable amount of time outdoors without protection from mosquitoes. To prevent ongoing transmission, targeting of groups, places, and activities with complementary interventions should consider setting-specific human behaviors in addition to epidemiological and entomological data.

Residual malaria transmission (also referred to as persistent or ongoing transmission) after high coverage has been achieved with core interventions such as long-lasting insecticidal nets (LLINs) presents a challenge to malaria control and elimination efforts [1]. Social patterns and human behavior may determine exposure to *Anopheles* mosquitoes and have an effect on transmission. To address residual transmission in a particular location, an understanding of when and where vector and human behavior intersect is therefore necessary [2–4].

In 2017, over 880 000 malaria cases were confirmed in Papua New Guinea (PNG) and it was estimated that 94% of the population was at high risk of malaria infection [5]. Since 2004, much progress has been made in reducing the malaria burden with financial supported from the Global Fund to Fight AIDS, Tuberculosis, and Malaria. The PNG National Malaria Control Program has achieved high ownership of LLINs and malaria prevalence below 1600 m altitude decreased from 11% in 2008/09 to < 1% in 2013/14 [6–9]. However, despite this success, malaria resurged dramatically across PNG by 2016/2017 with an estimated 8.6-fold increase in prevalence in only 3 years [8]. While certain shortfalls in the National Malaria Control Program coincided with this increase, use of LLINs had remained stable at around 50% since 2011 [8]. Early and outdoor biting of *Anopheles* mosquitoes had been identified as a threat to the effectiveness of LLINs. The peak exposure time for infective *Anopheles* bites shifted from later than 9 pm in 2008 to between 6 and 7 pm in 2011 [10, 11]. In light of such findings, human behaviors such as sleeping patterns, and social, cultural, and economic activities during early evening and nighttime, could increase exposure to infective mosquito bites.

Alongside the recent general resurgence in malaria, different trends have been observed in a number of sites in PNG [12]. Considering this within-country heterogeneity in malaria [12–15] and the cultural diversity, it is likely that different human behaviors are relevant for malaria transmission across the country [4, 16, 17]. Social and cultural patterns, livelihoods, and the response of people to specific interventions, together with local political and economic realities, have been responsible for changes in malaria epidemiology in the past [18]. For instance, travel and trade, clay collection for pottery, interarea marriages, bird of paradise hunting, gardening, sago harvesting, salt collection, and road and transport developments have been identified as social aspects influencing the malaria epidemiology in PNG [18–20].

This study aimed to better understand the role of human behavior in relation to malaria transmission and transmission heterogeneities by: (1) identifying activities and livelihood aspects potentially relevant for malaria transmission, (2) understanding which measures are currently being used to prevent or reduce mosquito biting in the study sites, and (3) identifying behavioral differences between population groups.

## METHODS

### Study Design

A mixed-method design was used to investigate human behavioral patterns with potential relevance for malaria transmission in 2 sites in PNG. Knowledge, perception, and practices related to malaria transmission, prevention, and sickness were also assessed. In-depth interviews (IDI), focus group discussions (FGD), a cross-sectional malaria indicator survey (MIS), and a population census were implemented in parallel.

### Study Sites

**Figure 1.**
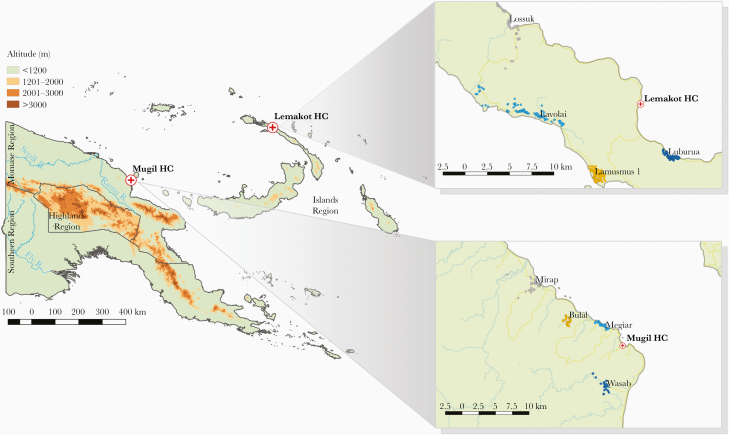
Location of the 2 study sites and the selected villages in each study site. Colored dots represent all identified households in the selected villages.

The study was conducted within the catchment area of 2 health facilities: (1) Mugil Health Center, Sumkar District, Madang Province and (2) Lemakot Health Center, Kavieng District, New Ireland Province (Figure 1). Villages with a high malaria burden at the health facility were selected for an exploratory visit. Following the visits, 4 villages were selected in each site based on accessibility and the explicit consent from the village leaders to participate. Two villages in each site included a scattered population distributed across a larger area while the 2 others included a population concentrated in a smaller area. The location of all selected villages can be seen in Figure 1.

#### Mugil Area

Mugil area is located in the rainforest area on the north coast of mainland PNG [21]. Cash crops such as copra (the dried meat of the coconut), cocoa beans, and betel nut (*Areca catechu)* are the main source of income. Selected villages in the Mugil area included 2 coastal villages (Megiar and Mirap) and 2 inland villages (Bulal and Wasab).

#### Lemakot Area

Lemakot area is located on the main island of New Ireland Province, which is long (approximately 200 km), narrow (approximately 8 km), and mountainous with a wet tropical climate [22]. Oil palm companies are well established in the area as a major provider of formal employment. The most important cash crops are oil palm fruit, copra, and to a lesser degree sago (*Metroxylon sagu*) and betel nut. The last 2 are grown for own consumption or to sell on a small scale within the community. Selected villages in Lemakot area included 2 west coast villages (Lamusmus and Lavolai) and 2 east coast villages (Luburua and Lossuk).

### Data Collection

#### Quantitative Data Collection

Quantitative data were collected during a cross-sectional MIS between September 2016 and October 2017. Adult household heads and all available residents (age > 6 months) of the selected households were invited to participate in the MIS. All data were collected electronically on tablets using the open-source platform Open Data Kit. One questionnaire was administered for each household (household questionnaire) and 1 for each available household member (individual questionnaire). Variables collected in the household questionnaire included size of the household, kinds of livestock in the household, type of water source, type of house structure, presence of window screening, type of toilet facility, education level of the household head, income source, perceived best malaria prevention methods, and ownership of LLINs in the household. Variables collected in the individual questionnaire included: demographic details (age and sex), use of LLIN the previous night, frequency of LLIN use, age of the used LLIN, the time the person went to bed the previous night (bedtime), sleeping place, history of antimalarial treatment in the last 2 months, and travel history in the last month. A population census in the 8 selected study villages preceded the survey. Household size, house coordinates, and age and sex of all household members were collected electronically using Open Data Kit. A photo of the house and its members was taken if participants agreed. A sample size of 80% of households was defined as the operational target to achieve a near-complete spatial representation of the study villages. The census and the MIS were conducted by a team of trained local field interviewers with previous research experience.

#### Qualitative Data Collection

Qualitative data on behavior, livelihood, and activities of different demographic groups with location and timing were collected using IDIs and FGDs. A local village representative was appointed by the village leaders to recruit study participants. Participants were selected to represent single and married men and women, with and without children, in the area. Inclusion criteria were age (age ≥ 15 years) and fluency in Tok Pisin (local lingua franca). Participants across all age groups were selected to represent younger and older demographics.

Explorations during the IDIs applied a time line follow-back method (TLFB). TLFB gathers behavioral information during a preselected time period. A calendar is used to structure the interview and assist the respondent’s recall strategy [23]. IDIs were guided by a calendar that covered everyday activities with particular focus on time spent indoors and outdoors during *Anopheles* biting times during the last 7 days. Two IDIs were conducted in each village (in total 8 per study site), 1 with a man and 1 with a woman. IDIs were conducted in Tok Pisin by a trained interviewer. IDIs were recorded, then transcribed verbatim and finally translated to English for the analysis.

Two FGDs (1 with men and 1 with women) took place in each selected village (in total 8 per study site). FGDs were conducted in Tok Pisin by a trained interviewer. FGDs were recorded and notes were taken by a second team member who was not the interviewer. Because the environment is a crucial part of understanding behavior, FGDs were accompanied by a hand drawn map on a flip chart. The map was based on a sketch (Supplementary Material 1) of the village developed by the community leaders. The sketch delineated the village according to the local community. On occasions, it included subdivisions of the village and or highlighted the landmarks (eg, houses, schools, plantations, swamps, etc.) considered important by each community. During the FGDs, the maps (Supplementary Material 2) added a sense of distance and time to the narrative. In addition, potential sites for transmission within or around the village were identified for each community. After data collection the interviewer reviewed and adjusted the notes while listening to the recording. Notes were used for the analysis.

IDIs and FGDs were conducted in parallel in each village. After completing the first round of 8 IDIs and 8 FGDs in 1 study site (Mugil area) re-emerging topics were identified and the collected data reached saturation without new topics emerging. The sample size was then established for both study sites.

### Data Analysis

#### Quantitative Data Analysis

Descriptive quantitative analyses were conducted using Stata/IC version 13.1 (Stata Corp). Responses from the household questionnaire were stratified by site and percentages with 95% confidence intervals (CIs) were calculated for each response. The hourly cumulative percentages of people sleeping, and people sleeping under a LLIN, were calculated for the period between 6 pm and 2 am for which data were collected. This information was used to graph the percentage of individuals protected under a LLIN for 0–2, 3–4, 5–6, and 7–8 hours during this time period. Full sleeping times curves (going to bed and wake-up times) were generated for adults based on responses of FGD participants. Responses from the individual questionnaire were stratified by behavioral groups (defined below) and percentages with 95% CIs were calculated for each site.

#### Qualitative Data Analysis

English transcriptions of the IDIs and the notes of the FGDs were analyzed independently by 2 researchers using a preestablished framework designed to identify activities and related information relevant to potential malaria transmission (time of the day, duration, location, and sex and age of the person or people reported for each activity). Coding differences were discussed and harmonized by the researchers coding the data. Consensus on coding was agreed after discussions. The analysis prioritized activities occurring between 4 pm and 8 am, 2 hours either side of dusk and dawn. Seven demographic groups exhibiting similar behavioral patterns (behavioral group) subsequently emerged from the framework, including: preschool-aged children (age ≤ 5 years), school boys (age > 5 and ≤ 16 years), school girls (age > 5 and ≤ 16 years), adult men in Mugil, adult women in Mugil, adult men in Lemakot, and adult women in Lemakot (age for all adult groups > 16 years). A composite profile for each behavioral group was then drafted, drawing on the respective IDI and FGD data for that grouping, to portray the most common activities and livelihoods with a special focus on potential exposure to mosquito bites. A further thematic analysis of the IDI and FGD data was also conducted independently by 2 researchers focused on identifying knowledge, attitudes, and practices towards malaria transmission and vectors, malaria prevention, and clinical episodes. Emerging themes were compared, discussed, harmonized, and added to the composite profiles. Saturation was reached with most IDIs and FGDs discussing the same or similar topics.

## RESULTS

**Figure 2.**
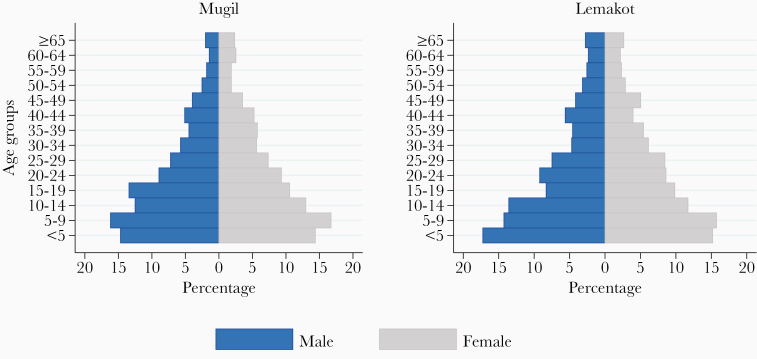
Age and sex distribution of the population in the study sites according to the baseline census.

The baseline census identified a total of 3364 individuals in the 4 villages in the Mugil area and 5470 in the 4 villages in the Lemakot area. Age and sex distribution of each study site are depicted in Figure 2. Women comprised 48% (1620/3364) of the population in Mugil and 47% (2888/5470) in Lemakot. The survey sample included 1927 participants in 398 households in Mugil and 1202 participants in 309 households in Lemakot. A total of 16 IDIs were conducted; the participants’ age ranged from 21 to approximately 55 years in Mugil and from 28 to 68 years in Lemakot. A total of 16 FGDs were conducted with 61 people aged between 15 and 61 years in Mugil and with 68 people in Lemakot aged between 19 and 76 years.

### Quantitative Data

#### Household Characteristics and Livelihood.

**Figure 3.**
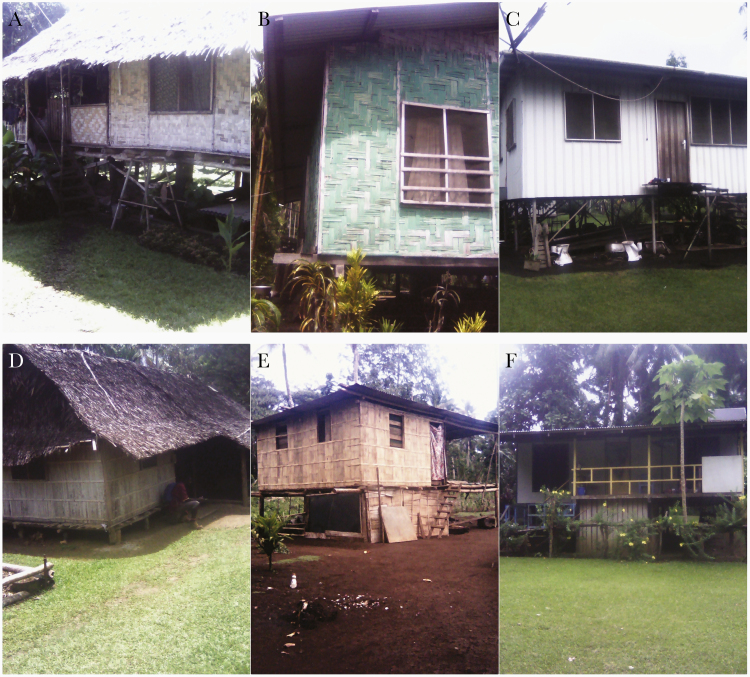
*A*–*C*, Examples of housing structures in Mugil: traditional structure (*A*), mixed (*B*), and modern (*C*). *D*–*F*, Examples of housing structures in Lemakot: traditional structure (*D*), mixed (*E*), and modern (*F*).

Table 1 includes key characteristics of households included in the MIS. In Mugil, more people lived in traditional houses than in Lemakot, where mixed construction was more common. Figure 3 exemplifies the types of housing in both study sites. The study considered traditional houses those constructed only with raw materials (roof, walls, and floor). Modern houses were constructed only with improved materials and mixed houses with both raw and improved materials. In Lemakot, the number of households with access to water at the dwelling (water tanks and piped) was considerably higher than in Mugil, where most households used surface water. The number of self-sustained households in Mugil was considerably higher than in Lemakot where more people were employed with a wage.

**Table 1.** Household Characteristics by Site

	Mugil (n = 398)		Lemakot (n = 309)	
Characteristic	Percentage	95% CI	Percentage	95% CI
Number of household members, mean	6.0	5.7–6.3	5.7	5.4–6.1
Households keeping animals				
Chicken	47	42–52	47	41–52
Pigs	51	46–55	49	43–54
Dogs	47	43–52	48	43–54
Cats	30	26–35	30	25–35
Housing type				
Traditional	74	68–78	47	42–53
Mixed	26	22–31	51	46–57
Modern	0	0–2	2	1–4
Window screening				
On all windows	25	21–29	19	15 -24
On some	37	33–42	34	29–39
On none	30	26–35	44	39 -50
No windows	8	6–11	3	2–6
Water source				
Surface water or well	79	75–83	34	28–39
Water tank or piped into dwelling	21	17–25	66	61–72
Toilet facility				
None	46	42–51	52	46–58
Outdoors latrine	53	48–58	47	41–52
Indoors toilet	1	0–2	1	0–3
Income source				
Regular wage	6	4–9	21	17–26
Self-employed	12	9–15	9	7–13
Self-sustained	81	77–84	67	62–72
Other	1	1–3	2	1–5
Educated household head	92	89–94	97	94–98
Achieved education				
≤6th grade	51	46–56	51	45–57
≥7th and ≤ 12th grade	45	40–50	38	33–44
Higher education	4	2–7	11	8–15

Abbreviation: CI, confidence interval.

#### Malaria Prevention.

Malaria prevention methods were reported in similar frequency by respondents in both sites. The use of mosquito nets was the prevention method mentioned most frequently in both study sites. Interestingly, 4 of the mentioned prevention methods were linked or related to cleanliness of self and around the house. A complete list of the perceived “best prevention methods” is available in Table 2.

**Table 2.** Best Malaria Prevention Methods According to the Respondent by Site

	Mugil (n = 398)		Lemakot (n = 309)	
Methods	Percentage	95% CI	Percentage	95% CI
Mosquito net	82	78–85	81	76–85
Remove rubbish	65	60–69	72	67–77
Clean the house	45	41–50	42	37–48
Clear grass around the house	45	41–50	39	33–44
Drain stagnant water	32	28–37	34	29–39
Burn leaves or husks	14	11–18	11	8–15
Stay in good health	12	9–15	13	10–18
Cleanliness	8	6–11	8	6–12
Mosquito coil	4	2–6	3	2–6
Herbs	2	1–4	0	0–2
Avoid mosquito bites	1	0–2	0	0–2

Abbreviation: CI, confidence interval.

##### LLIN Ownership and Use.

The majority of households in both study sites owned at least 1 LLIN (Table 3) but households in the Lemakot area owned fewer nets and people used them less consistently than in the Mugil area.

**Table 3.** LLIN Ownership and Use by Site

	Mugil		Lemakot	
Indicator	Percentage	95% CI	Percentage	95% CI
Reported LLIN ownership (Mugil, n1 = 398; Lemakot n1 = 309)				
At least 1 LLIN	100	98–100	93	90–94
1 LLIN for every 2 people	72	68–76	61	57–65
Reported used LLINs (Mugil, n2 = 1554; Lemakot, n2 = 971)				
LLINs used the previous night	70	67–72	34	31–37
Number of people sharing a LLIN				
1	43	40–46	43	38–48
2	33	30–36	29	24–34
3	18	15–20	23	19–28
4	5	4–7	4	3–7
5	1	1–2	1	0–2
Reported not used LLINs (Mugil, n3 = 471; Lemakot, n3 = 637)				
Reasons for a particular LLIN not being used				
Spare LLIN	81	77–84	49	45–53
Too hot	6	4–9	26	22–30
Damaged LLIN	5	3–7	2	1–3
User away	4	3–7	4	2–6
Other	2	1–4	7	5–9
No mosquitoes	1	1–3	2	1–3
Don’t know	0	…	10	8–13

Abbreviations: CI, confidence interval; LLIN, long-lasting insecticidal net; n1, number of households; n2, total number of reported LLINs; n3, reported number of LLINs NOT used the previous night.

**Figure 4.**
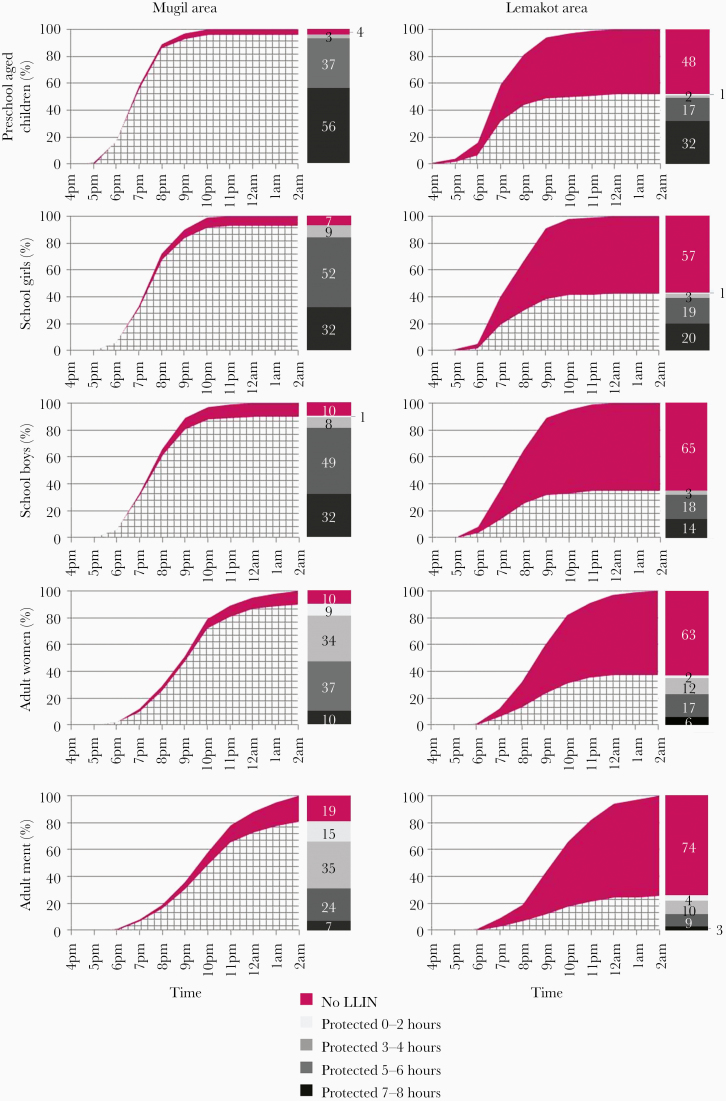
Sleeping times and net use for each behavioral group by site, sleeping under a long-lasting insecticidal net (LLIN; net pattern) and sleeping without a LLIN (solid). The bar and color code represent the number of hours (0–2, 3–4, 5–6, and 7–8) protected under a LLIN between 6 pm and 2 am.

Overall, LLIN use was 89% (95% CI, 88%–91%) in Mugil but only 37% (95% CI, 35%–40%) in Lemakot. The difference between the 2 sites was found in all behavioral groups (Figure 4). In both sites, preschool-aged children were the group with the highest LLIN use: 96% (95% CI, 93%–98%) in Mugil and 52% (95% CI, 44%–59%) in Lemakot. In contrast, adult men used LLINs the least: 81% (95% CI, 77%–84%) in Mugil and 26% (95% CI, 21%–31%) in Lemakot.

In both sites, 1 LLIN was used, on average, by 1.9 people (Mugil, 95% CI, 1.8–1.9; Lemakot, 95% CI, 1.8–2.0); most nets were occupied by 1 to 3 people (Table 3). The most common reason for not using a particular LLIN was that the net was considered “spare,” that is it was reported to be saved for later use, either for a new house, a visitor, or a particular person who was absent at the time of the survey [24]. In some households, certain LLINs were considered “spare” even though not all household members were sleeping under a net. In Lemakot, a considerably higher percentage of LLINs were not used because it was considered too hot (Table 3).

##### Bedtimes and protection by LLINs.

In both sites, individuals reported going to sleep later the older they were. Adult men, who were also least likely to use a LLIN, went to sleep the latest. In Mugil and Lemakot, 80% of preschool-aged children had gone to sleep by 8 pm (86% using a LLIN in Mugil and 44% in Lemakot) and 80% of school girls and school boys by 9 pm (84% and 81% using an LLIN in Mugil and 39% and 32% in Lemakot, respectively). In both sites, adult women went to sleep earlier than adult men. In the Mugil area, 80% of women had gone to sleep by 11 pm (81% using an LLIN) and 80% of adult men by midnight (73% using an LLIN). In the Lemakot area, 80% of women had gone to sleep by 10 pm (31% using an LLIN) and 80% of adult men by 11 pm (22% using an LLIN) (Figure 4). Less than 10% of respondents reported sleeping outdoors in both study sites.

Waking-up times of adults assessed during FGDs revealed that people wake up between 3 am and 9 am in Mugil and between 4 am and 8 am in Lemakot. In Mugil, adult women appeared to wake up earlier than men (Supplementary Material 3).

Despite a high LLIN-use reported by all behavioral groups in the Mugil area (81% and more), the differences in the time people go to bed lead to large variation in the time a person was protected by an LLIN (Figure 4, bars). For example, while 56% of preschool-aged children were protected by an LLIN more than 6 hours between 6 pm and 2 am, this was the case for only 7% of adult men. FGD waking-up data from Mugil (Supplementary Material 3) suggest that women may be at a slightly higher risk of early morning exposure due to earlier waking-up times. Similar differences in time protected by a LLIN were observed in Lemakot even though the effect of the time when people go to sleep under an LLIN is comparably less relevant due to the overall low LLIN use (Figure 4).

Recent travel within and outside the province was reported by 9% (95% CI, 7%–10%) of people in Mugil and 10% (95% CI, 8%–12%) in Lemakot. In both sites, adult men were most likely to have travelled. Recent intake of an antimalarial was reported for 8% (95% CI, 7%–9%) of people in Mugil but only 4% (95% CI, 3%–5%) in Lemakot. Only in Mugil, children were more frequently reported to have taken an antimalarial than adults (Table 4)

**Table 4.** Self-reported Sleeping Habits and LLIN Use, History of Recent Travel, and Recent Use of Antimalarials by Behavioral Group and Site

Group	Slept Indoors, % (95% CI)	Slept Under LLIN, % (95% CI)	LLIN Every Night, % (95% CI)	Travel in Previous 30 d, % (95% CI)	Antimalarials in Previous 60 d, % (95% CI)
Mugil	96 (96–97)	89 (88–91)	77 (75–79)	9 (7–10)	8 (7–9)
Preschool-aged children	98 (96–99)	96 (93–98)	90 (86–93)	5 (3–9)	10 (7–14)
School girls	100 (100–100)	93 (89–95)	83 (79–87)	5 (3–8)	11 (8–15)
School boys	99 (97–100)	90 (86–93)	78 (73–82)	6 (4–10)	9 (7–13)
Adult women	98 (96–99)	90 (87–92)	77 (73–80)	9 (7–12)	5 (3–7)
Adult men	90 (86–92)	81 (77–84)	63 (58–67)	14 (11–18)	6 (4–9)
Lemakot	92 (90–93)	37 (35–40)	24 (22–27)	10 (8–12)	4 (3–5)
Preschool-aged children	97 (94–99)	52 (44–59)	36 (30–44)	7 (4–11)	3 (1–6)
School girls	98 (94–99)	43 (36–50)	29 (23–36)	5 (3–9)	5 (3–10)
School boys	94 (89–96)	35 (29–43)	21 (16–28)	7 (4–12)	4 (2–8)
Adult women	94 (92–96)	37 (32–42)	24 (20–29)	11 (8–15)	3 (2–6)
Adult men	80 (75–85)	26 (21–31)	15 (12–20)	15 (11–20)	5 (3–8)

Abbreviations: CI, confidence interval; d, days; LLIN, long-lasting insecticidal net.

### Qualitative Data

The 7 composite profiles (1 for each behavioral group) constructed from the IDI and FGD data are presented in full in Supplementary Material 4–10. A summary of the 7 profiles, highlighting common and distinct activities, potential malaria transmission risks, and preventive actions by site is presented below. The following section initially describes general behaviors and livelihoods identified throughout all behavioral groups and time periods. Activities carried out by specific groups across 5 relevant time periods (predinner, dinner, postdinner, morning, and weekends) by site follow the initial summary. The time periods were added to simplify the narrative and offer a temporal dimension. However, the timing of events within and between households was occasionally more flexible than the narrative and activities could extend through time periods.

**Figure 5.**
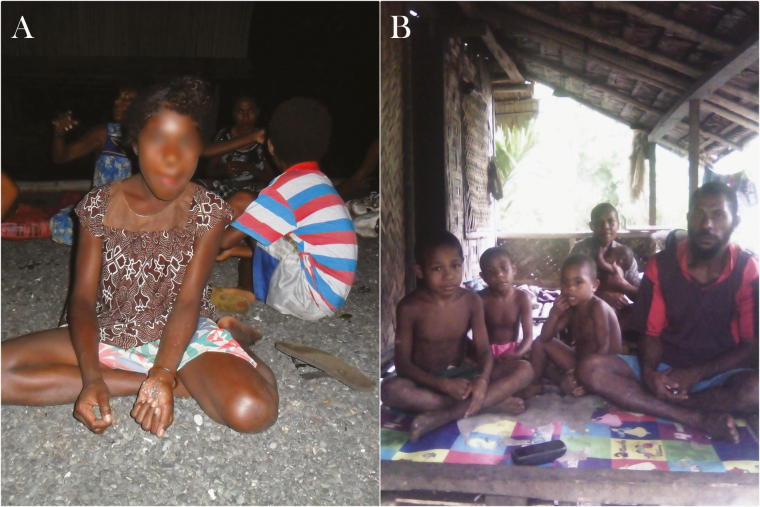
*A*–*B*, Garments commonly worn in Mugil area by children and adults. *A*, Outdoor gathering at night and betel nut chewing. *B*, Veranda space where people commonly spend evenings.

**Figure 6.**
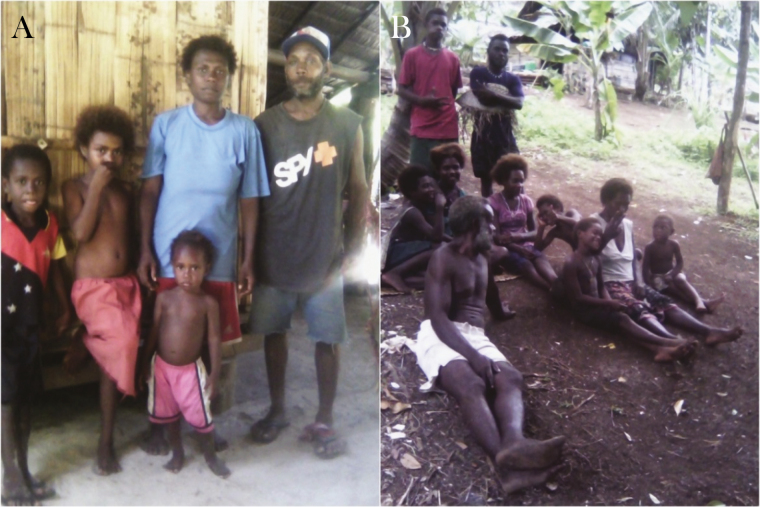
*A*–*B*, Garments commonly worn in Lemakot area by children and adults. *B*, Outdoor space cleared of vegetation for the family to gather using mats to sit on the ground.

**Figure 7.**
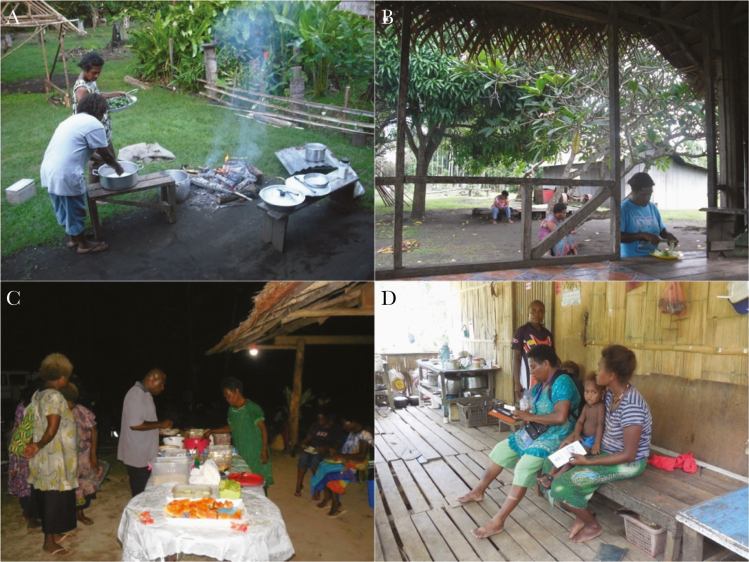
*A*, Outdoor cooking in Mugil (2016). *B*, Outdoor spaces in Mugil (2016). *C*, Night gathering, extraordinary celebration in Lemakot (2017). *D*, Outdoor sitting and cooking spaces in Lemakot (2017).

Outdoor activities between dusk and dawn, absence of outdoor prevention for mosquito biting, lack of protective clothing (eg, long sleeves and long pants), and open structures accessible to mosquitoes for gatherings and sleeping were identified as factors conducive to malaria transmission in both study sites. Clothing and footwear were similar in both sites, during day and night and among all behavioral groups. Commonly worn garments, such as shorts and short-sleeve t-shirts left arms, legs, and feet exposed to mosquito biting; the torso of men and children were commonly exposed as well. The most common footwear were thongs (flip-flops) and in many occasions people walked barefoot (especially children). While at the water, women and girls were usually wrapped in a laplap (sarong) or a towel while boys and men were wearing only a pair of shorts. Babies were usually naked. Open areas accessible to mosquitoes were very common for private and public gatherings (eg, dining area and church). Figure 5, Figure 6, and Figure 7 depict clothing and open spaces in both study sites. Using smoke to scatter mosquitoes was the only reported method to repel mosquitoes outdoors in both study sites. The use of topical repellents was absent and mosquito coils were rarely used. Nighttime activities occurring outdoors and gathering static groups of people included: cooking, eating, chatting, selling, watching TV, watching live sports (at the field), drinking alcohol, smoking, chewing betel nut, doing homework, playing, and praying. Most of the spaces where people gather in the village were open or semiopen without major physical barriers and mosquitoes may freely enter and exit such places or structures. Specific activities carried out by different groups in each study site and their relevant aspects for potential exposure to mosquito biting are described in detail in the section below.

### Mugil—Activities, Potential of Exposure to Malaria Mosquitoes, and Preventive Measures

#### Predinner Period (4 to 6 pm)

At 4 pm on a typical working day, preschool-aged children in the village play outdoors while waiting for the school children to return from school and adults from the market or the “blocks” (cash-crop plantation). The school children arrive at the village at 4 or 5 pm depending on how far they have to walk from school (usually between 30 minutes and 2 hours). They follow the main road and once at the village they follow the walking paths. Once at home, school girls look after their younger siblings and help with chores, mostly outdoors close to the house. After school, boys help in the garden or the blocks at the outskirts of the village; they collect betel nut or bananas and carry them to the house. Men and women return to the village at 4 or 5 pm. Women return from the food garden or the market, men from the block. They all go to the river and bathe. Most people bathe or wash twice a day, once early in the morning and once before dinner. They walk to a stream or the river bank 5 to 10 minutes away. The girls go with their mothers and younger siblings to bathe and fetch water. Women and girls combine bathing times with other water related chores like doing the laundry or washing the dishes. Such additional chores prolong up to 90 minutes the time women spend by the water. Once at the river, the boys swim and play before going back to the house. Men and boys spend more recreational time at the water, not only bathing but also swimming or fishing. Areas for males and females at the river banks or streams are separated. Women, girls, and small children are back at the house at 5 pm or 6 pm. Then, women cook with the help of their children. The kitchen is usually outdoors or partially open (Figure 7).

All activities between 4 pm and 6 pm occur outdoors and many of these activities occur by the water. The timing and place of these activities seem conducive for malaria transmission especially when the duration of the activities extends until it is dark. Such an open environment easily exposes the community to mosquito bites. Bathing, doing the laundry, cleaning the dishes, fetching water, and swimming are activities likely to convey potential exposure risk because they take place close to the water. In addition, the walking paths to and from the garden, school, or water bodies are likely to represent a risk because those paths are transited daily and connect the scrubland to the village, resulting in ideal place to encounter mosquitoes [25].

#### Dinner Period (6 to 8 pm)

Dinner takes place between 6 and 8 pm and the whole family gathers outdoors, either on the veranda or in an open space next to the kitchen (Figure 5 and Figure 7). Dinner is usually followed by drinking tea, chatting, and betel nut chewing and is accompanied by a smoking fire. The smoke of a fire with coconut husks or leaves is the most common method people use to repel mosquitoes. Dinner conveys potential exposure risk because it occurs outdoors and it gathers the family in a static activity in an unprotected space with arms, legs, and feet exposed to mosquito bites for a considerable amount of time. In addition, the youngest of the children and babies usually fall asleep outdoors and stay exposed up to 2 hours before they are taken to their sleeping place.

#### Postdinner Period (8 pm to Bedtime)

Different groups (men, women, and youths) gather on different days of the week for praying fellowship. Young men gather after dinner. Depending on the season they go spear fishing [26] or hunting at night or they watch a sports match or movies on the available television screen in the village. They also chat, listen to music on a mobile phone, chew betel nut, and smoke at the market stands by the road. When the battery of the phone runs out, they look for places where they can charge it and then wait (usually outdoors) while the phone charges.

Most people sleep indoors in shared rooms and mostly on the floor on a mat or a thin mattress. Preshool-aged children go to sleep by 8 pm, school boys and school girls by 9 pm, women by 11 pm, and men are the last going to sleep by midnight or even later (as reflected in the quantitative data; Figure 4). Women usually share the net with 1 young child. School girls usually share the net with 1 other girl and school boys with 1 other boy. When boys reach puberty they start sleeping alone; if nets are available they sleep under one, otherwise they do not. Generally, adult men also sleep alone under their own net.

Evening activities, like the praying fellowship, convey potential exposure risk because the activities gather people in open or semiopen spaces; in addition, people walk to and from the venue without any protection. Boys and men seem to be at higher risk of exposure because they are awake longer than their female counterparts. Activities like chewing, smoking, and watching TV after dinner are a potential risk of exposure. Once asleep the main potential risk is sleeping without a LLIN.

#### Morning Period (Waking Time to 8 am)

During the weekdays the women are the first waking up (4 to 5 am) and prepare breakfast. School boys and girls wake up at 6 am to go to school. School starts at 8 am and children often need to start walking before 7 am. Before going to school, some boys collect firewood or cut the grass around the house. Girls fetch water and wash the dishes from the previous night. Men and preschool-aged children are the ones waking up the latest (7 am and later). For school children, the potential exposure could be associated with the distance to school; the greater the distance the earlier they rise, shower, and walk, therefore the longer the potential exposure time.

Toilet facilities are scarce in the area with 46% of the households having no access to a toilet facility (Table 1). People in the area mainly use the ocean, the bush, or an outdoors latrine (when they have access to it). Walking distance to the closest toilet area varies between 50 and 800 m. The use of the toilet occurs across all time periods but mostly before washing in the morning and afternoon. Stagnant water is commonly found close to the toilets, potentially exposing people to mosquito bites.

#### Weekend and Seasonal Variation

During the weekend, everybody in the house wakes up later (7 am) than during the weekdays. The family has breakfast at around 8 am. The afternoon is similar to the weekdays. The family has dinner together between 6 and 8 pm. In general, the family stays awake until later. Sometimes, the whole family joins a communal movie screening in the village. Screening areas are often open. Visiting or receiving visitors is more common during the weekend. The extended family gathers and moves around within the village and to neighboring villages. Saturday is the common day to go get supplies at the closest town (Madang). The first bus passes by at around 8 am; everyone going to town needs to wait by the main road shortly before 8 am. On Sunday, people prepare for church service, which usually starts at 9 am; therefore, people wake up and bathe between 7 and 8 am. At 6 or 7 pm, the praying fellowship starts. Most of the family members join the prayer. The prayer finishes at 9 pm. The families then walk home and go to sleep at about 10 pm. During the weekend, potential exposure during the morning period is likely to be reduced because people wake up later than during the week. Conversely, they also stay awake until later, potentially increasing exposure at night.

When the family plans to grow a new garden or block, school boys help to clear out the area after school. They work a couple of hours in the afternoon and walk back home during or shortly after sunset. Households grow more than 1 kind of crop resulting in multiple planting and harvesting seasons a year. During harvest season for coconut and cocoa beans, boys and young men take overnight turns to supervise the drier for a small fee. People are more active in the gardens and blocks during planting and harvesting season, making it more likely that they work until dark and increasing potential exposure to mosquito bites

Volleyball and soccer teams (young men and women), assemble and train for the local tournaments a few times a year. They usually train between 4 and 6 pm at the school or the church grounds. Sports convey potential exposure, especially for boys, because they gather and chat close to the fields after practice, and the timing coincides with anophelines biting times. In contrast, girls go directly home after training. School holidays could also pose potential risk, especially for boys because they stay awake and outdoors for longer periods at night.

### Lemakot—Activities, Potential Exposure to Malaria Mosquitoes, and Preventive Measures

#### Predinner Period (4 to 6 pm)

A typical afternoon in Lemakot is very similar to Mugil. School children return home from school and adults return home from work. At home, people engage in chores and bathing. Girls and women perform household chores including laundry, washing dishes, and cooking. Boys and men work at the garden, cut the grass, or collect food, betel nut, or firewood. Young men play rugby. People wash themselves twice a day, in the morning and in the late afternoon or evening. People commonly wash at home, next to the water tank or water drum. The availability of water tanks, public taps, and wells is higher in Lemakot than in Mugil area, therefore people walk shorter distances to a water source. During the rainy season, families with a water tank share the water with their neighbors, resulting in people fetching and storing water closer to home. During this period, all activities occur outdoors and many of them close to the water. Activities like bathing, doing the laundry, washing the dishes, and swimming are likely to convey potential exposure to mosquitoes, especially when they extend in duration until dark. However, greater access to protected water sources might reduce contact with mosquito breeding sites and might reduce potential exposure to mosquito bites.

#### Dinner Period (6 to 8 pm)

Dinner takes place between 6 and 8 pm, as in Mugil, and is accompanied by a smoking fire to repel mosquitoes. The families sit outdoors and eat. After dinner, people drink tea and chew betel nut or smoke. During dinner, exposure is associated with the extended period of time families spend gathered and static outdoors.

#### Postdinner Period (8 pm to Bedtime)

Recreational activities such as watching movies or sports matches on a screen, chatting and chewing betel nut, or religious events (fellowship prayer), are common after dinner. However, the dynamics slightly change every 2 weeks when wages are paid. (Over 20% of the households in the Lemakot area receive their main income from paid employment; Table 1.) Firstly, a larger number of road stands are set up and opened for longer hours. They commonly sell snacks, food, soft drinks, and other recreational commodities like alcohol, betel nut, and cigarettes. A great number of people, especially men, gather outdoors by the stands or the road to drink, chew, and chat during the night. Alcohol consumption is a common practice that may continue until the early hours of the morning. Hence, after dinner the potential risk is mainly related to recreational activities. In Lemakot, activities resulting from the constant influx of cash in the community could increase potential risk of exposure during the weekends every 2 weeks, starting on Friday night.

People sleep mostly indoors in a shared room and on the floor using a mat or mattress. Most women are sleeping by 11 pm and men by 12 pm. The number of people sleeping under a mosquito net is significantly lower in Lemakot compared to Mugil area (Figure 4). Hot nights seem to increase the potential risk of exposure because people are more likely to sleep outdoors and more reluctant to sleep under a LLIN. People reported perceiving the air under the LLIN to be hotter and damper.

#### Morning Period (Waking Time to 8 am)

People in Lemakot generally wake up earlier than in Mugil with most of them awake by 6 am. Women and men seem to rise at the same time, in contrast to Mugil where men sleep longer. Plantation employees work shifts from 6 am to 2 pm. Therefore, transport circulates before 6 am for the plantation workers. People walk towards the main road as early as 5 am, hence many of them wake at 4 or 5 am and spend a considerable amount of time outdoors and unprotected while anophelines are still active.

Most of the toilet facilities available are outdoors or nonexistent; 52% of the households in the area reported not having access to a toilet (Table 1). People walk between 50 and 800 m to a latrine or a toilet area in the bush or at the beach, leading to potential exposure to mosquito bites.

#### Weekend and Seasonal Variation

The weekend dynamics are similar to those in Mugil; people tend to wake up and go to sleep later. Entire families work at the garden or the block during the day. Visits to the main town (Kavieng) are usual during the weekend. People wait for the bus at the main road earlier than 8 am. Fishing is a time-consuming activity. Mostly men go to the sea for hours returning in the afternoon and cleaning the fish at the beach during sunset. Night fishing [26] is also a common practice in the area, especially at times when the fish are scarce during daytime. The harvesting of sago is more common in Lemakot than in Mugil. Men and women go to the sago swamp for a whole day, potentially increasing exposure to bites when times at the swamp start before 8 am or finish after dark. Recreational activities like family gatherings in the late afternoon and night are more common than during the week. Other common nighttime activities include religious gatherings and movie screenings. Recreational and religious activities at night are likely to convey risk of exposure because people gather in open and/or semiopen spaces.

People in the Lemakot area grow different kinds of crops with multiple planting and harvesting seasons throughout the year. Clearing of wild vegetation, planting, and harvesting are likely to increase the risk of exposure because these activities prolong the time people spend in the planting areas and postpone walking back to the village until the early evening. Once a month with the new moon, people living in the villages between the swamp and the beach (especially on the east coast) collect mud crabs. The harvest takes place while the crabs move from the swamp to the beach [26]. Collecting the mud crabs is likely to increase exposure to mosquito biting during this period because it occurs outdoors, close to the mangroves, and at dusk.

Once a year, a big festival known as Malangan happens for 3 days and 3 nights and the whole community gathers to celebrate. The preparations for the feast take months with people gathering regularly in the afternoons. Feast preparations include “mumu” (steaming in an earth oven), a cooking tradition that happens overnight and requires hours of outdoor preparations (digging the hole, heating up the stones in a fire, preparing the meat and vegetables, burying the food for a long cooking time, and unearthing it once cooked). Events like funerals gather people outdoors at night. The community (in the Lemakot and Mugil areas) usually meets at the house of the deceased and pays respect to the family. The mourning usually continues day and night until the burial. Depending on the circumstance, time to the burial could take up to a week. Extraordinary activities, such as the Malangan and funerals, are likely to result in higher risk of exposure to mosquito biting because they extend until night and take place outdoors.

## DISCUSSION

This study highlights the substantial amount of time people spend outdoors or in open structures, and with clothing that offers little protection against mosquito bites, in 2 rural settings of PNG where mosquitoes frequently bite outdoors and early in the evening [11]. Between dusk and dawn, people in both study sites are engaged in activities likely exposing them to mosquito bites. Potential exposure may be linked to specific activities that vary to a certain degree between different behavioral groups identified in this research. Sleeping under a LLIN was the only nontraditional prevention method identified in both study sites, yet with very different coverage levels in Mugil (89% use) and Lemakot (37% use).

Bathing, washing laundry and dishes, swimming, fishing, hunting, harvesting sago, and collecting mud crabs are everyday outdoor activities close to or by the water during anopheline biting times. Bathing, washing laundry and dishes, and swimming are activities that take place within the village as opposed to harvesting sago, hunting, collecting mud crabs, and fishing, which occur at the swamp, at the beach, in the bush, and on the ocean or a river. Because most people consistently return from their gardens or blocks to the village before dark and do not usually stay there overnight, exposure to *Anopheles* mosquitoes is more likely to happen on the way rather than in the plantations and gardens. In contrast to some practices in Asia and Africa [27, 28], people reporting a second house to sleep at the garden or plantation were the exception.

Considering the abundance of water bodies in the study areas (and, in fact, large parts of PNG) outdoor activities after dusk are likely to result in exposure to mosquitoes [19, 29–31]. Social and cultural activities after dusk include sporting events (local games or on shared television), religious activities (regular church services and praying fellowships or seasonal retreats or festivities), funerals, and cultural festivals (eg, Malangan). Potential seasonal exposure may be linked to farming activities. Planting and harvesting occur a few times a year depending on the crops grown by a household. Most reported outdoor activities have previously been identified as potential risk for malaria transmission in other settings [32–36]. In PNG, a large sporting event held over several days in Sandaun Province in 2017 was followed by a major malaria outbreak (personal communication, Dr Kelebi, Provincial Health Authority).

While outdoor activities may expose people to mosquito bites, organized activities and regular events may also provide an opportunity for implementing targeted control measures. However, this study found little evidence of reliable forms of outdoor mosquito biting prevention between dusk and dawn. When outdoors, the only preventive measure consistently reported across quantitative and qualitative data was producing smoke to repel mosquitoes. Keeping the house and its surroundings clear of vegetation, water, and rubbish were considered good measures to prevent malaria in both study sites, according to the quantitative data. When indoors, sleeping under an LLIN was the only method consistently reported to prevent mosquito bites and malaria. Behavioral and livelihood elements such as little skin coverage by clothing, no use of mosquito repellents, minimal use of mosquito coils, and open housing structures could exacerbate and maintain malaria transmission despite the use of LLINs. While the potential of LLINs to reduce malaria morbidity is well known [37, 38], inconsistent or low use (eg, in Lemakot) limits their effectiveness and may lead to differential impact of this intervention in different sites [12]. The ownership and use of LLINs found in this study coincide with previous regional findings of net use in PNG. Higher ownership and use have been consistently reported in Madang Province (Mugil area) compared to New Ireland Province (Lemakot area) [6, 39, 40]. In this study in Lemakot, less than 30% of school children (boys and girls) and adults (men and women) reported sleeping under a LLIN every night. In comparison, over 60% of all age groups reported sleeping under a LLIN every night in the Mugil area. A common reason for not using LLINs in the Lemakot area was the notion that it is too hot to sleep under them. Despite similar temperature range in both sites [19, 41, 42], this notion is rarely described in the Mugil area. A more consistent use of the LLINs seems to diminish the “too hot” perception suggesting that LLIN users need to adapt their perception before consistently using the LLIN.

The study also highlights limits to the protection offered by LLINs, especially for adults, complementing previous findings from PNG and elsewhere [3, 10, 36, 43]. For instance, despite a high LLIN-use in the Mugil area (90% for adult women and 81% for men), in the 8-hour period between 6 pm and 2 am 50% of the men and 43% of the women were under the protection of the LLIN for 4 hours or less. According to the available wake-up data (Supplementary Material 3), the majority of adult men and women reported being awake before 6 am in both study sites, suggesting additional potential exposure in the early morning. Moreover, previous studies in PNG and other settings identified outdoor exposure in the early hours of the evening and in some settings early in the morning [10, 44–46], highlighting the need for complementary interventions offering outdoor protection. In addition, commonly applied binary LLIN-use indicators expressed as a percentage of people using a net the previous night may be a poor reflection of the protection offered to different age groups at different times of the night.

While sleeping outdoors was not very common, a clear distinction between indoor and outdoor spaces was difficult to apply in many households in the study sites as house structures and building materials often do not present major physical barriers to mosquitoes.

The considerable amount of time spent outdoors presents a window of potential exposure to malaria-carrying mosquitoes. Because LLINs primarily prevent indoor biting, complementary methods to LLINs are needed to prevent outdoor biting in the evenings and the morning. Potential options include vector control measures like larval source management, topical and spatial repellents, and attractive toxic sugar baits [46–48]. However, an essential feature of understanding human behaviors is the potential to target places, groups, and activities. In the study sites, places where people regularly gather at night, such as churches or movie screening areas, could be targeted for group prevention, either treating surfaces with insecticide or using spatial repellents. In contrast, when people roam widely at night or early in the morning, as men and plantation workers do, then personal protection (eg, topical repellent or more protective clothing) may be more appropriate. Specific groups (eg, boys, men, and plantation workers) could be targeted with behavioral change communication to encourage more protective clothing choices at night and early in the morning. Given the current lack of outdoor prevention and the potential of malaria exposure, ensuring that effective treatment is readily available at all health facilities is of paramount importance.

This study identified 7 population groups based on specific patterns of activities: preschool-aged children, school boys, school girls, male adults in Mugil, female adults in Mugil, male adults in Lemakot, and female adults in Lemakot (Figure 4, Table 4, and Supplementary Material 4–10). Age and sex were important factors determining human behavioral patterns. The main event drastically changing a child’s behavior is starting school. Once in school, behavioral differences between boys and girls become evident. Girls’ chores are more house oriented (eg, fetching water, cooking, doing the laundry, or washing the dishes) while boys have less house-oriented chores (eg, chopping wood, cutting the grass) and more freedom to spend their time with peers and away from the house. Once out of school the range of activities carried out by men and women are affected by their environment. In this case, the kind of cash crop and availability of wage employment in each site considerably change livelihoods in the communities. In Lemakot, the regular influx of cash prolongs the risk of exposure to *Anopheles* bites for informal vendors and customers during pay weekends every 2 weeks. Previous studies already associated some activities with specific population groups: household chores like fetching water and laundry have been associated with women while drinking and socializing with men and sports with adolescent boys [32, 34]. In general, adult men appeared to remain outside for the longest and be least protected by LLINs, which could be due to women being more likely to share their LLIN than men.

Subnational heterogeneity in malaria is a result of a variety of factors and human behavioral differences are likely to be one contributing factor. Understanding local human behaviors may help to identify risks of exposure to malaria mosquitoes and opportunities for targeted control interventions. However, assessing the contribution of human behavioral patterns to ongoing malaria transmission requires the consideration of local entomological data and information on infection prevalence in humans. Ongoing analyses by the study team aim to combine findings from entomological and prevalence studies with data described in this manuscript to understand when and where malaria transmission really happens.

Our study was not free of limitations. Recall bias may have affected the data collected, especially with IDIs. It is possible participants could not recall all relevant activities carried out the previous week, in the exact time, order, and place they occurred. When discussing annual events and seasonality, some participants struggled recalling annual activities and the exact month when they occur. FGDs were conducted with participants of different ages because the topic was not considered to be very sensitive. However, it is possible that during the discussions young participants were intimidated by the senior members of the group, limiting younger people’s inputs in the discussion. In order to prevent this, the interviewer tried to actively integrate every participant in the discussions. The qualitative analysis was framed around activities and did not allow for a deeper thematic analysis that could raise other unforeseen relevant issues (eg, gender or cultural dynamics). Quantitative data on the number of hours spent under a LLIN did not include wake-up times because this information was not collected during the MIS. An approximation of wake-up times was derived from FGDs as contextual information. Bedtimes and wake-up times (FGDs) were self-reported, and there was no way to ascertain the accuracy of the reported times. However, the consistency of bedtime patterns reported in the MIS and FGDs in this study and the congruence with previous findings [10] provide confidence of the validity of the reported times. Finally, the study did not include any prevalence of malaria or local entomology data in the analysis; therefore, it assesses potential exposure to mosquito bites rather than risk of malaria infection or actual exposure. An in-depth analysis of the prevalence distribution in the study sites is provided elsewhere (D. Rodriguez-Rodriguez, A. Ross, Salib M, et al. unpublished).

Our findings highlight the potential of outdoor exposure to mosquitoes to hamper malaria control and elimination efforts as people spend a remarkable amount of time outdoors without using any form of protection against mosquito biting. Particularly in setting as diverse as PNG, control programs should consider local knowledge of setting-specific human behaviors to target groups, places, and activities with complementary interventions to accelerate efforts towards malaria elimination.

## Supplementary Data

Supplementary materials are available at *The Journal of Infectious Diseases* online. Consisting of data provided by the authors to benefit the reader, the posted materials are not copyedited and are the sole responsibility of the authors, so questions or comments should be addressed to the corresponding author.

jiaa402_suppl_Supplementary-Material

## Notes

***Acknowledgments.*** The authors thank all participants and communities who agreed to participate in this study. We thank the Papua New Guinea Institute of Medical Research field team and acknowledge the support of the National Department of Health through Mr Leo Makita and his team. We are also greatful to John Bosco Keven who kindly provided the picture in Figure 5 panel A.

***Financial support.*** This work was supported in part by the Special Programme for Research and Training in Tropical Diseases, grant number WCCPRD4426109 2016/639607 to Papua New Guinea Institute of Medical Research for data collection. WHO Ethics Review Committee approval number: ERC.0002671.

***Supplement sponsorship***. The supplement is sponsored by TDR, the Special Programme for Research and Training in Tropical Diseases, based at the World Health Organization.

***Potential conflicts of interest.*** All authors: No reported conflicts of interest. All authors have submitted the ICMJE Form for Disclosure of Potential Conflicts of Interest. Conflicts that the editors consider relevant to the content of the manuscript have been disclosed.
