# Supplementary material for: Human Behavior, Livelihood, and Malaria Transmission in Two Sites of Papua New Guinea
Source: J Infect Dis. 2021 Apr 27;223(Suppl 2):S171–86. doi: 10.1093/infdis/jiaa402 (PMC8079136; doi:10.1093/infdis/jiaa402)
Supplement: jiaa402_suppl_Supplementary-Material [file jiaa402_suppl_supplementary-material.docx]

## Supplementary Data

### Supplementary data 1


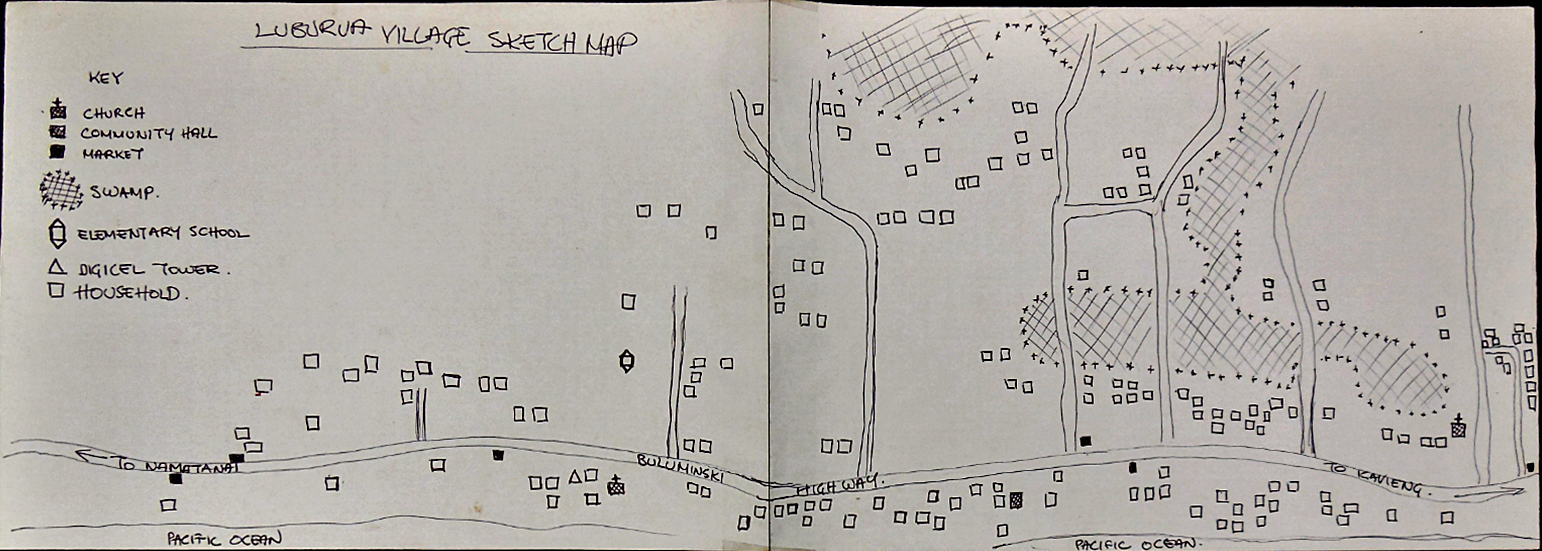


Sketch Example

Sketch of Luburua (Lemakot area) as provided by the village leaders.

### Supplementary data 2


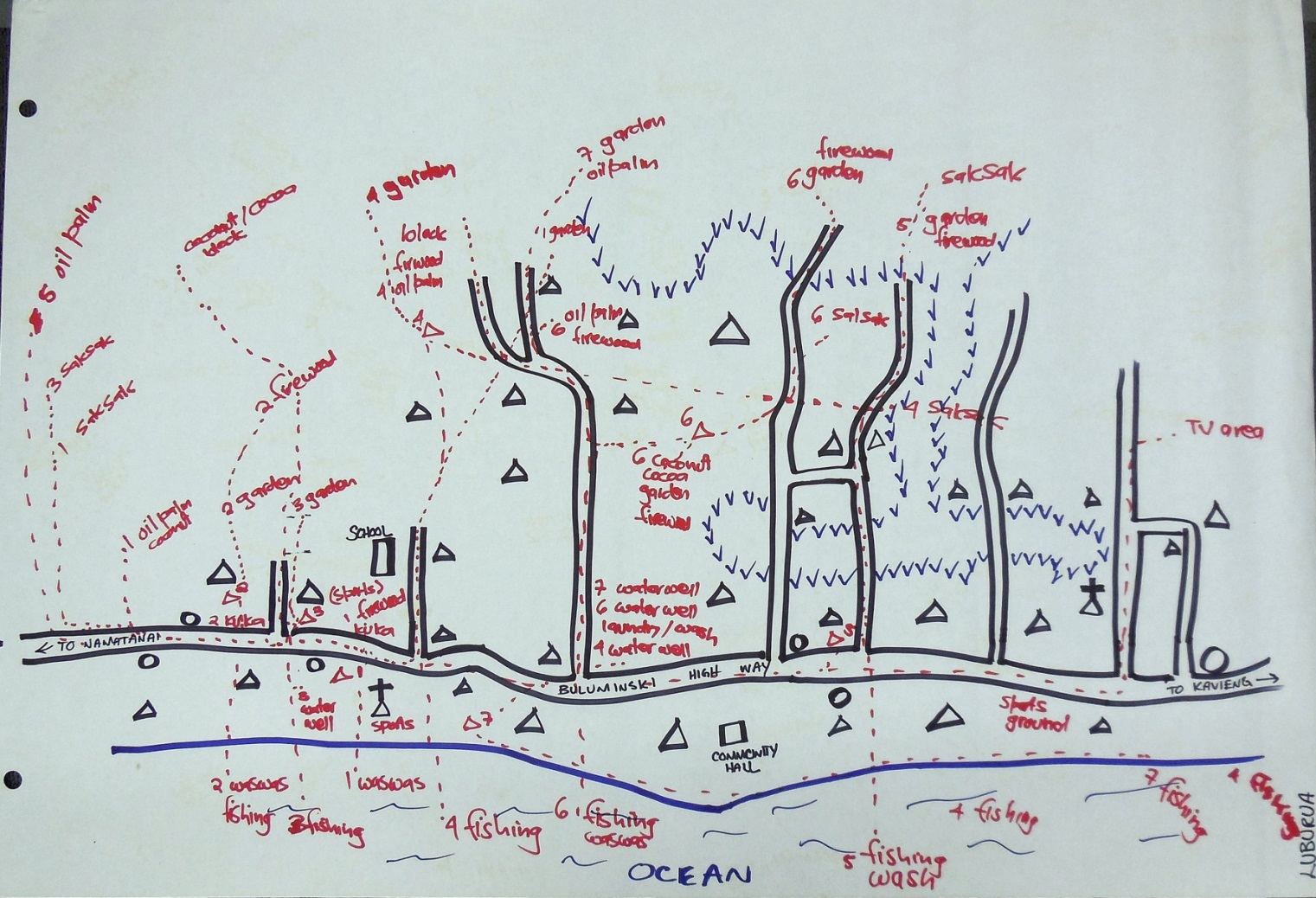


Map of Luburua used to accompany the FGD with women. In black the village characteristics, in blue the swamp and ocean delineation and in red the location of the reported activities.


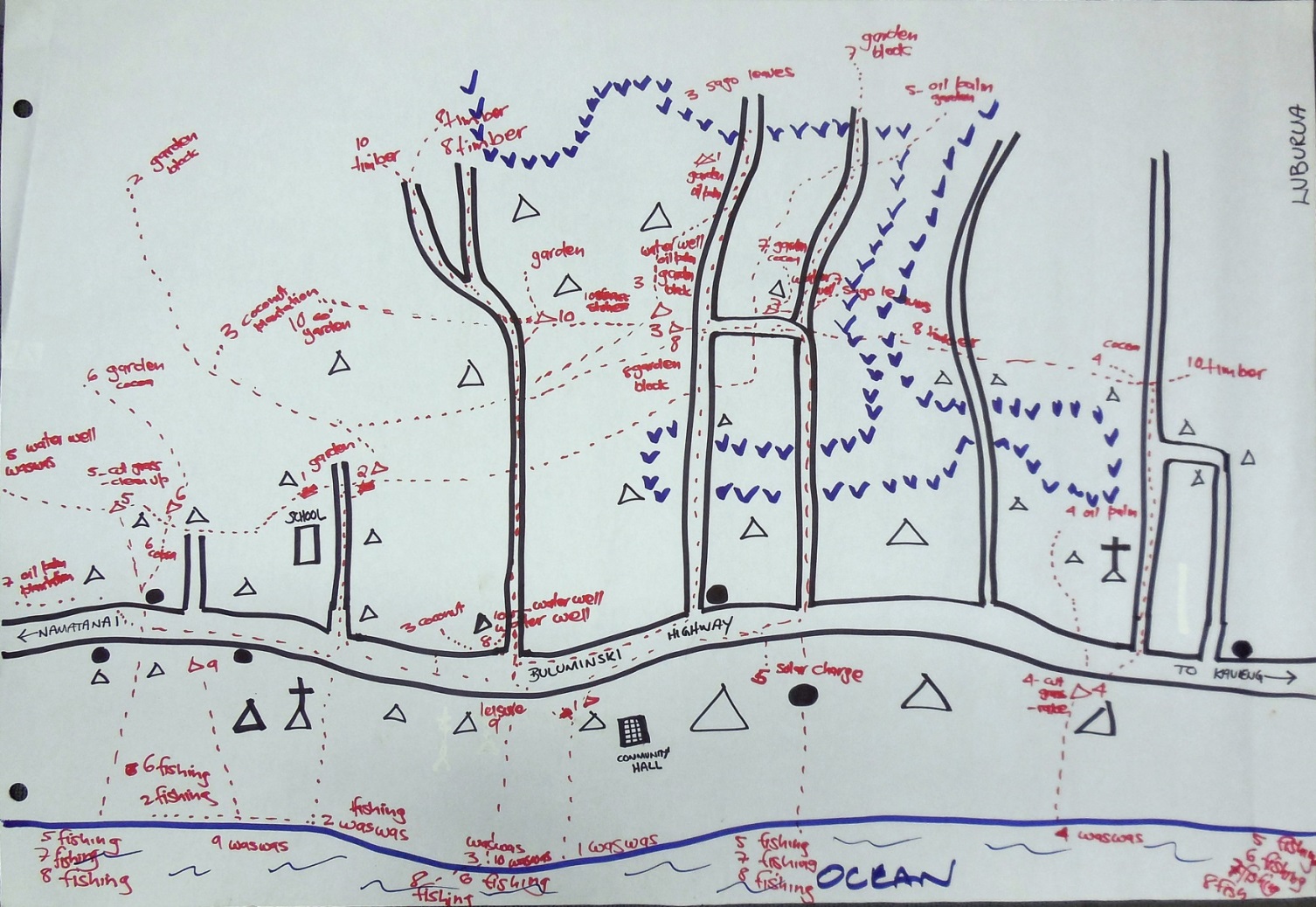


Map of Luburua used to accompany the FGD with men. In black the village characteristics, in blue the swamp and ocean delineation and in red the location of the reported activities.

### Supplementary data 3


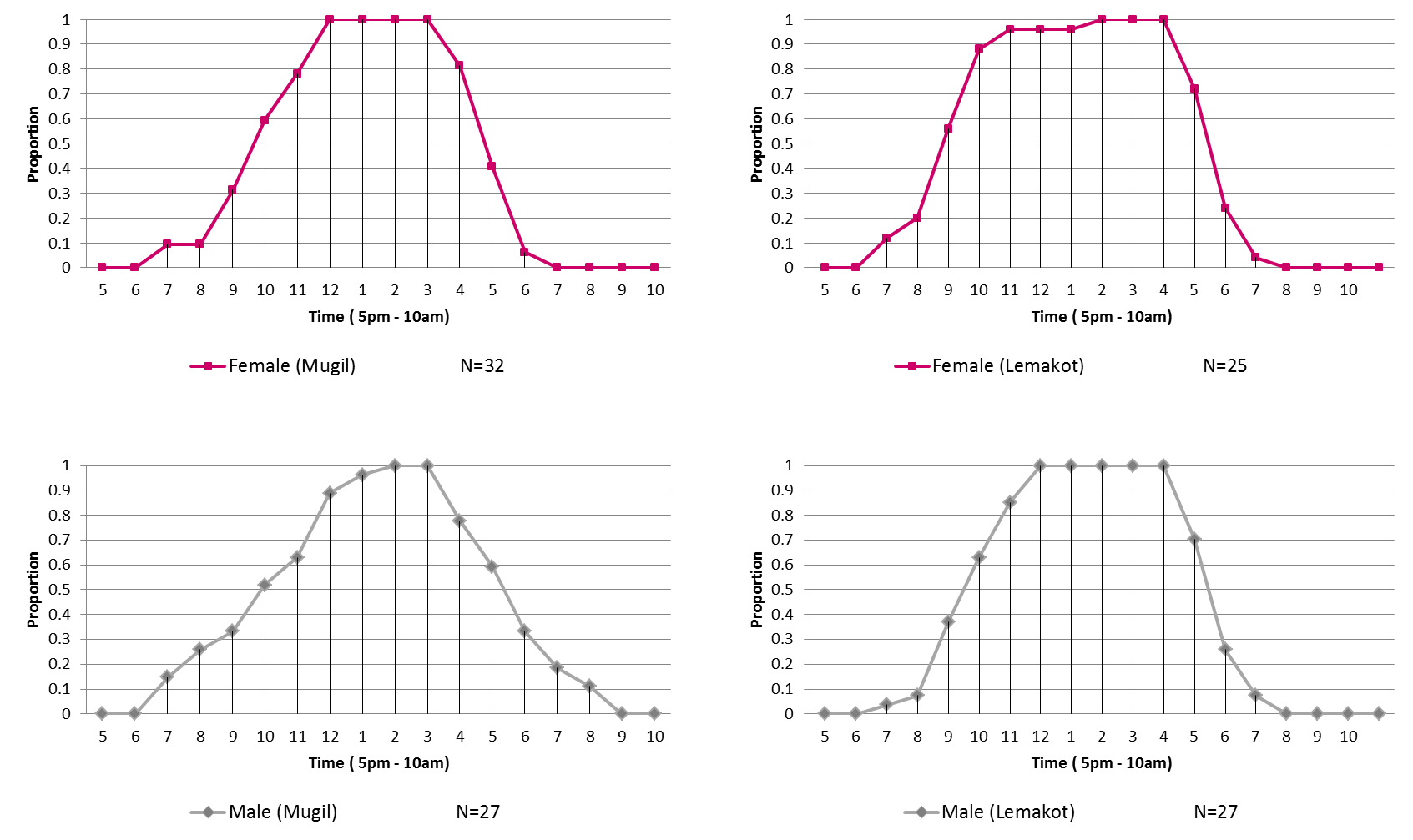


Sleeping times (going to bed and waking-up times) collected during Focus Group Discussions in both study sites for adult males and females. N denotes the number of responding FGD participants

### Supplementary data 4: Child- Serah, 4 years old

During the week Serah is the last waking up in the house. Her parents and older siblings wake up earlier to get ready for school but as a young child Serah sleeps until 7am. She usually shares the sleeping space and mosquito net with her mother and her baby brother. During the hot nights she sleeps wearing only underpants. She wakes up calls for her mother or older sister and they will take her to use the outdoor latrine. They usually walk 200m to get there, and it is a quick visit that usually takes 10 minutes. When they are back at the house her sister dresses Serah and her little brother. Serah wears a skirt or a pair of short. She only wears a shirt when it is rainy and cold. Her little brother is usually only wearing a cloth nappy. Both of them are always barefoot. Once dressed Serah and her brother sit outside close to the kitchen and play until breakfast is ready at about 8am. She eats breakfast with her mother and her younger brother who like her is not in school age yet. They spend most of the day together following their mother around, playing and learning the house chores. Serah as a girl imitates her older sisters and wants to help cooking, sweeping and fetching water while her baby brother collects pieces of wood and helps raking around the house. After breakfast they play around the house until their mother finishes cleaning up after breakfast. Then they follow her to the river or the beach where they bathe and accompany their mother while she does laundry and dishes. This usually takes one or two hours. After the river they return to the house if Serah feels tired she takes a nap in the kitchen on a mat her mother unrolls for her and her baby brother. During the day they do not sleep under a net. Sometimes they sleep in the room; in any case the longest they nap is about an hour. After her nap she eats a little something and plays around with the kids next door. Sometimes when her mother needs to do some gardening Serah accompanies her to the garden. If she is tired and her older siblings are around she stays with them or her grandmother at the house. During the late afternoon she awaits for her siblings in school they come back at 4pm and spend time with her. They take Serah and their baby brother to the river and bathe them before dinner. They leave at around 5pm and come back at 6 or 6:30pm. Her family usually eats dinner at 6 or 7pm during the week. They eat together and they all sit outside. After the food they drink tea and chat, around 8pm Serah gets sleepy. Sometimes she falls asleep, for half an hour, on the veranda. Her big sister would take her in the room when she goes inside. Other times, when her mother is also tired she would take the two youngest children into the house and put them to sleep. Her baby brother and she usually share the bed and mosquito net with their mom.

The weekends are different since her older siblings do not go to school. They all wake up later and spend more time together. They go fishing or swimming to the beach or the river. They go at around 10am and come back in the afternoon. Sometimes the whole family goes to the garden and harvest food for the week. They go early so they can work before the sun reaches the hottest point. At noon they rest and continue working in the afternoon when it cools down. They usually have a long dinner on Saturday. Serah stays up longer and her arms and legs are exposed to mosquito bites since her clothing does not cover them. Her family cannot afford mosquito repellent to apply on the skin but they usually make a fire with the intention of dispersing the mosquitoes around the house. On Sunday they all go to church service together. It usually starts at 9 am and lasts till noon. They have to bathe at 7am to be on time. After church they all go home have lunch and rest. At 6 or 7pm the praying fellowship starts. On Sunday most of the family including Serah join the prayer they walk to the church area and back. It takes about 20 minutes since they walk slowly and chat. They wave away the mosquitoes with their hands if they feel them on the way to church. The church is a semi-open with screened windows but during the prayer the door remains open. They usually finish at 9pm. The family walks home and go to sleep shortly before 10pm.

### Supplementary data 5: Male School Child – Tom, 15 years old

Tom wakes up at 6am. He is the oldest of his siblings therefore he stopped sharing a bed net and mattress with his younger siblings. He usually sleeps on a mat in a shared bedroom. He does not use a net. He wakes up at 6am. Since his family does not have a toilet facility, he goes to the close by bush first thing in the morning. He leaves the house wearing only the shorts he slept in, his arms and legs quite exposed to mosquito bites. Sometimes he collects fire wood on his way back; it takes him some extra 10 minutes to collect the wood. Once a week he cuts the grass around the house it takes him about 15 minutes. Afterwards he goes to the river bank and bathes. He walks 10 minutes to the water and bathes in less than 10 min. His body is shortly submerged under the water. He is mostly standing with the water up to his waist. He comes back home shortly before 7am and wears his school uniform (a pair of shorts and a button short sleeve shirt). He prepares his lunch and eats breakfast before leaving the house. At about 7am he starts walking to school it takes him 45 to 60 min to get there. School day starts at 8 am and ends at 3 pm. The school year runs from January to December and it has four terms. Tom has school break in April, June, September and December). Tom does not travel, not even during holidays therefore he spends them in his village just like during the weekends. Tom and his classmates hang around at school after class. They chat and make plans for the weekend. Then he walks back home. He is back at 4:30. At home he changes his uniform to his working shorts and sometimes a t-shirt. Depending on the day of the week he might need to go to the family garden and collect betel nut to sell or bananas for dinner and breakfast. During dry season and if the family plans to grow a new garden or block Tom joins his brothers and clear out the area. They work a couple of hours in the afternoon and walk back home during or shortly after sunset. The way to the garden follows parts of the main road. The main road is unpaved and the tire tracks they pass are good *Anopheles* breeding sites. At home he grabs his towel and goes to river to bathe and swim. Often he meets his friends at the river and they play together in and out of the water, it is usually dark by the time he goes home. Once at home he eats dinner with his family at around 7pm. They all sit outside and usually make a fire. They add the coconut husks to the fire to produce smoke and repel the mosquitoes. After dinner Tom takes some time to do his homework on the veranda under the only light available in the house. When he does not have school work he stays around the fire talking to the rest of the family and chewing betel nut. He goes to bed at 10 or 11 pm. Some nights if there is an important Rugby match on TV; Tom meets with his friends to watch. The viewing area is covered from the rain but it has no walls. On Friday; they watch movies until roughly midnight. If there is nothing to watch the boys chat and listen to music at the selling-stand by the road. Some of his friends have phones and they gather together to listen music and find a place where they can charge the phone. Whenever a generator is on in the village they ask if they can use the plugs. They sit around the fire and wait until the battery is charged. Tom walks back home and sleep at 1 am or so. He does not own a mosquito-net since his younger siblings use the available ones and he feels sleeping under the net is too hot in any case.

During the weekend Tom wakes up at 9 or 10 in the morning. He eats breakfast and right after he meets with his friends at the beach. Sometimes they play rugby or volleyball games. Other times if any of the boys needs help with a big task they work together and get bush material for building a house. When they need sago palm leaves for the roofing they go to the swamp to get them for two or three hours after breakfast. When he wakes up before 9am he goes to the garden with his family and helps her mother carry the food back to the village. In the afternoon, he would bathe at the river and have dinner at home. He then meets his friends. He knows the village very well and he has a lot of freedom to move around and meet with the other boys. They watch a movie if any is screening in the village if not the boys gather at the road and buy betel nut. They spend the night talking, chewing betel nut and listening to music. He gets back home passed midnight and goes to sleep. On Sundays if he wakes up early he goes to church service at 9 am with his family. After service, the family eats together at 4 or 5pm. After the meal they have tea and sit around the fire. If Tom is tired he goes to sleep at about 10 pm otherwise he stays up and chats with the others or he finishes his school work before going to sleep at 11pm or midnight. His family never uses mosquito repellent since its use is unknown to them, it is not available in the local store and in town it is too expensive for his family to purchase it regularly.

### Supplementary data 6: Female School Child – Kati, 10 years old

Kati sleeps with her older sister; they share a net and a thin sponge mattress on the floor. She sleeps on a t-shirt and a pair of shorts. Day and night her clothes exposed her arms and legs, she never wears socks and her shoes are a pair of flip-flops. She wakes up at 6 am she uses the outdoor latrine first thing in the morning. The latrine is about 100 meters away from the house it takes her less than 10 minutes to go there and back. Immediately after she goes to the river and bathes. A little stream runs downhill from her house, she usually goes there when she has pots to wash from the previous night. When there is water left in the drum she bathes by the drum and gets dress in her school uniform at the house. She wears a mid-leg skirt and a short sleeve shirt to school. Once ready she helps her mother prepare breakfast. Shortly before seven in the morning she starts walking to school. She walks together with her siblings it usually takes an hour to get there. They follow the main road but when they are lucky they get a ride from a passing car that they know and they get early to school. School day starts at 8 am and continues until 3 pm. When school day is over; she walks back home with some of her friends. She is back home at 4 pm but some of her friends; the ones living further from school; walk an extra hour to get home. Once at home she changes from her school uniform (to either a skirt or shorts and a t-shirt) and helps with the house chores. First she goes to fetch water mostly at the river but one neighbour has a big tank that is closer than the river. During rainy season they can get water from the tank more often than when rain is scarce. In any case the river is a 10 min walk away. When she is back her mother starts cooking. Kati helps to peel the vegetables or scrape the coconut. They seat on two little stools in the outdoors kitchen. When she is done with the cooking she grabs her towel and goes to the river. She washes her own laundry twice a week. She sits on a rock by the river bank with her feet in the water when she does her laundry. When she finish she bathes her little siblings and herself. They come back home after 30 to 40 min at the river. They walk back home between 6 and 7 pm. Then the whole family has dinner together. They sit outside next to the fire. They have an outdoor space were they are protected from the rain but there are no walls. Those days with a lot of mosquitoes her family throws coconut husks or tree leaves into the fire to repel the mosquitoes with the smoke. After dinner depending on the day of the week she accompanies her mother to the evening prayer at the church grounds. They usually go at 8 pm. They walk 20 min to the church. They have a small flashlight to light the way there and back. If she has school work she stays home and finishes her homework on the veranda. In very rare occasions a mosquito coil is available to repel the mosquitoes. Insecticide commonly referred to as “Mortein” is available on the local store. Once or twice a year, when too many mosquitoes disturb their sleep her father sprays the rooms to kill the mosquitoes in the sleeping areas. Unlike her brothers she spent most of her free time at home helping her mother with house chores and babysitting her youngest siblings. She usually goes to sleep before 10pm.

On the weekends she wakes up at 7 or 7:30 in the morning. She uses the latrine, eats her breakfast and goes to the river. She bathes and does the laundry with her mother and sister. The three of them spend over an hour at the river bank. They come back home hang up the laundry and she helps her mother with the house chores. Sometimes if there is need for planting or harvesting they go to the garden where they grow the family food or to the block where they grow the cash crop. The block is further away. They usually walk for an hour or an hour and a half. The leave the house at about 10am. Once in the garden they work for three or four hours with a break in between. When they finish they walk back to the village. Some weekends they go fishing for most part of the day. They usually get back at 4 or 5pm. Back at home they rest for an hour before preparing dinner. Kati helps with the cooking as usual and at 6 pm or 7pm they have dinner. Some nights she visits her aunts and stays for an hour or so. At their place sit outside chat and chew betel nut. Her aunts live close by just a few minutes walk down the road. Other times she brings food to her grandfather, he lives alone so her family looks after him. In rare occasions her whole family joins a movie screening at the neighbours. She only stays until 9 pm. At this time her mother takes Kati and her little siblings back home. Her dad and her big brother stay until late. On Sunday she wakes up at 7am she goes to the river, bathes and washes the dishes then she prepare for Sunday school and church service. Sunday school starts at 8 am, one hour before the mass. After the mass the family goes back home and she helps prepare dinner. As usual all the cooking and eating takes place outdoors. They all eat together at 4 pm. They drink tea and they chat, they chew betel nut and rest. Kati prepare her school work for next week before going to sleep.

### Supplementary data 7: Adult male (Madang) – John, 23 years old

John is not married and he lives with his two older brothers. Their house is close to their parent’s house, they live independently but they share the latrine. John and his brothers share a sleeping room and each of them has a mosquito net. Most nights they sleep under the nets and they sleep wearing a pair of shorts and no shirt. John wakes up at 7am and uses the outdoor latrine 100 m away from the house. Right after, he collects some wood in the garden behind the house and starts a fire in the outdoors kitchen. At about 8am he cooks some bananas directly on the fire and makes tea. Afterwards, the three brothers go to the river and bathe then they dress in shorts and a t-shirt. Their legs and arms are exposed to mosquito bites day and night. They never wear socks and their shoes are a pair of flip-flops. They walk to the coconut and cocoa block were they work. It takes them 30 to 40 minutes to get there. They follow a narrow walking path and they have to cross a pair of small streams on the way. Depending on the amount of work that needs to be done and the season the time they spend at the block varies. They either clear the block, plant new trees or harvest. On harvest season they collect coconuts and dry them under the sun. Once they are dried they compact them in sacks and arrange transport to pick them up. There is no big plantation or company in the area, therefore they have to arrange the sell within the community or go to Madang town and sell there. They also harvest and sell the betel nut. Buyers from the highlands regularly come with big trucks to buy sacks of betel nut and transport them to the highlands. When they harvest the cocoa beans they sell them green within the village since they do not have a drier to dry and store the beans. These are the three main cash crops they grow in the area. Close to the block they have a garden where they grow their own food. After working in the blocks or the garden for two or three hours they go to the beach close by at noon. They take shelter from the strong sun for a couple of hours. They swim and take a nap or rest at the beach before going back to work or fishing. In the afternoon if they don’t have betel nut to chew they collect it from the block before returning to the village. During a couple of months of the year John trains for the volleyball or soccer tournament. Every afternoon from 4 to 6 pm he goes to the field and train with his team mates. The training area is outdoors in the church grounds 150m away from a stream downhill. After training he goes back to the house grabs his towel and goes bathe at the river. He stays about 30 min at the river bank with his feet in the water until he gets hungry and goes home. John cooks something if there is nothing ready at the house. Otherwise he eats dinner and then leaves to meet his friends. During the right season he and his brothers dive in river at night and spear-fish [26]. They spend most of the time submerge or with their bodies under the water but on the way to the river and back their torso, arms and legs are exposed to mosquito bites. Most nights when John is not diving and someone is playing something on a screen he watches. Screens to watch are usually in open areas like verandas. When there is nothing to watch he gathers with his friends at the road to chew betel nut, chat and listen to music. He walks back to the house after 11 pm and goes to sleep. John knows malaria is transmitted by a specific kind of mosquito. That is one of the reasons he sleeps under the mosquito-net. However when he is outdoors he is not very concern of getting bitten by mosquitoes but he is annoyed by them.

During the weekends he wakes up at 9 or 10 am he uses the outdoor latrine, lights a fire and drinks tea sitting outside. Sometimes when he has business in town he walks to the road gets in a bus and goes to town (Madang). He spends a few hours there buying supplies. He gets back to the village in the afternoon using the same bus. In the afternoon he rests a bit and naps at the beach. Other times he goes to the garden to plant and harvest. He also checks on the block and collects betel nut. Some Saturdays he goes fishing to the sea with one of his brothers, they go for some hours in the afternoon; they get back at around 4pm and clean the fish at the beach before walking back to the house at around 5pm. At night he walks around the village with his peers. Sometimes they watch a rugby match on TV, sometimes they chew betel nut, smoke brus (dry tobacco leaves) and - if they have money –they buy beer. They gather outside for hours sometime around a fire sometimes just under the light of a selling stand at the road. They stay up till passed midnight. On Sunday morning he gets ready for church. He wakes up, uses the outdoor latrine, bathes at the river and has breakfast before 9am. Then he joins the church service at 9 am. In the afternoon he joins his family, they usually have a gathering at his parent’s house where they all eat, have tea and chat for hours sitting outside around the fire. Like most families in his village the only measure they take to repel mosquitoes is the smoke from the fire. When John feels tired he walks to his house. If he is hungry he has another cup of tea and biscuits or chews betel nut again while sitting on the veranda. He listens to music in his phone before going to sleep. If his phone runs out of battery he walks around and finds a place to charge it; sometimes at a house with a generator or a solar panel power source. He walks back home after his phone is charged and sleeps passed midnight.

### Supplementary data 8: Adult female (Madang) – Jenny, 48 years old

Jenny is a mother of five. She sleeps with her youngest daughter and they both share a LLIN. She wakes up between 5 and 6 am every day. Her family does not have a toilet therefore she walks 800m to the beach area commonly used as a “toilet” by women in the village. Twenty minutes later she is back in the house. She cooks breakfast for the school children in the semi-open kitchen. Once the older children leave to school she sweeps around the house and cleans up the kitchen area. She collects all dirty dishes and pots and goes to the river with the youngest children. She walks to the river at 9 or 10 in the morning and she spends one hour there with the children. They stay by the edge of the river sitting on a big rock. She washes the dishes and pots first. Then she bathes herself and the children. Some days of the week instead of dishes she takes the laundry to the river. In any case she walks back home and gets dressed before noon. She usually wears a skirt or a laplap (wrap) with a shirt or a meri-blouse (long loose blouse worn by women in PNG) but when she goes to the garden she wears garden-duty clothes (shorts and T-shirt). Her feet are always exposed since she do not wear socks and her shoes are a pair of flip-flops . Once dressed, she prepares to go to the garden or the market depending on the day of the week. When no one is around to babysit the little children she takes them with her otherwise they stay home with their older siblings or their grandmother. Early in the week she would go to the market with the food the family harvest during the weekend. To go to the market she follows a small road that eventually connects to the main road it takes her 45 to 60 min to walk there. If a car passes by and she knows the owner they give her a ride on the open back of the car and drop her at the main road. Once at the main road she walks further or gets on the bus depending on which market she goes to: the village market is close by but Kubugam market the big market in the area is further away so she takes the bus. Later in the week she goes to the garden and harvests food for dinner and breakfast. She walks 30 min to the garden and crosses a few small creeks on her way there specially during rainy season. Some areas get easily flooded. The family diet changes according to the season and the availability of food at the food-garden. Jenny also helps maintain the blocks of cocoa and coconuts twice a week. The blocks belong to her family and are far from the village. To get there she walks 90 minutes to 2 hours, she follows a narrow walking path between blocks to get there. In the afternoon she returns back home between 3 and 5 pm. When she gets home she rest a little and waits for the school children to get back from school, they are usually back at 4:30pm. They all go together to the river and fetch water and bathe; the trip tales up to 45 min. When they are back Jenny cooks dinner. When dinner is ready at about 6pm Jenny calls out the family and she serves the food. They all eat together, drink tea and chew betel nut until dark. They sit outdoors and eat in an open area next to the fire. They use the fire smoke to try to repel mosquitoes. Twice a week she goes to the prayer fellowship at 8 pm if she is not too tired. She walks to the church area followed by some of her children. They walk 20 minutes to get there. If they feel mosquitoes around they use a small towel and wave it around their legs and arms to try to prevent mosquito bites. The church is a semi-open space with mosquitoes freely flying in and out. When the prayer is over, the family walks back. The latest Jenny goes to sleep during the week is 11 pm.

During the weekend she wakes up a bit later since the children do not have school. On Saturday morning she wakes up, prepares breakfast and then gets ready to go to the garden with the children, they all go together to carry the food back from the garden. Some Saturdays they go fishing they go to the beach with line and hook and fish there. Jenny stands on a submerge rock close to a reef and fishes for hours with the water up to her waist. If Jenny still has food or betel nut to sell from the week she goes to the market. She gets back in the afternoon at 4 or 5pm and rests before cooking dinner. On Saturday her eldest daughter visits and brings her grandchildren. She looks after them while cooking and when dinner is ready they all eat together nest to the fire. Whenever she has someone visiting they stay up till late, chatting, chewing betel nut and drinking tea. She goes to sleep passed midnight until all visitors leave. On Sunday she wakes up at 7am. She send all the children to the river early and only after they are all back and having breakfast she goes herself and bathes. The family walks to the church area for service at 9 am. They stay in church until midday when they walk back home. She starts cooking right away since her extended family usually gathers on Sunday. Sometimes they gather at her house some other times they gather at another house but they all contribute with the meal, either they cook together or they bring a prepared dish. When the meal is ready they sit outside, eat and talk for hours. When it gets dark the family disperses and walk back to their home. Jenny makes sure the school children get ready for the week and she has tea before going to sleep at 10 pm. Jenny is afraid of malaria, especially of her children getting sick. When they get a fever she treats them at home but if they do not improve she know she need to go to the health facility and that expense could be catastrophic for the family.

### Supplementary data 9: Adult male (New Ireland Province) – Andrew, 51 years old

Andrew shares the sleeping area with his wife and the children. But unlike them he does not have a LLIN. He sleeps wearing a pair of shorts and no shirt. During the week he wakes up at 6 or 7 am. First thing in the morning he goes to the beach, to the area men usually use as “toilet”. It takes him 20 minutes to walk there and back. He grabs his towel and go bathe next to the shared well behind his house. He usually wears shorts and a T-shirt and his arms, legs and feet are exposed to mosquito bites. If needed, he cuts the grass around the house or chops wood for the fire; it takes him 30 minutes to complete each task. Once finished, he has breakfast and goes to the work in the block where he is growing coconuts and oil palms. The oil palm block is far from the house he walks 60 minutes to get there. He follows the main road, most of the blocks around the area are surrounded by an ample road that allows the trucks to drive around and collect the oil palm harvest. The road is full of tire tracks and puddles that are very suitable *anopheles* breeding sites. He and his family harvest the oil palm block every two weeks during harvest season. The rest of the year he clears the block area, works in his food garden or collects and dries the coconuts. When he has enough dry coconuts to sell he goes to the nearby big plantation where once a week a local buyer weights the sacks and buys them from the farmers in the area. He spends most of his day in the blocks but he takes a break at noon when the heat is the strongest. In the afternoon he walks back to the village and goes to the river. After his bath he has dinner with the family, they all sit outside and dine at 6 or 7pm. When his wife is not around he starts the fire and cooks some bananas. Once a week there is a prayer fellowship for men, he joins one or two times a month, the meeting starts at 8pm and finishes at 9pm. He walks 20 min to the church area. Other nights he goes for a walk at 9pm before going to sleep. He smokes and chews betel nut with the neighbour or other peers that he meets during his walk. If there is a rugby game on TV he goes watch the match. A venue in the village shows all the big matches. The venue is semi open and mosquitoes fly around. Andrew is the last person going to sleep in the house. He goes to bed at 11 pm or midnight.

During the weekends he wakes up at 8 or 9 am. When the wind and the sea are calm he goes fishing. He spends three or four hours at sea. He mainly fishes using a line and hook but other men in the area also go diving and collect sea cucumbers or spear fish. When fish is scarce during the day they go fishing at night. Saturday is also the day he goes to Kavieng to buy supplies if they are needed. The days he goes to town he wakes up earlier since he has to walk 30 min to the main road to catch the bus. He takes the bus at 8 am gets to town at 10 am purchases the goods and returns to the village with the afternoon bus. Saturday afternoon he stays home and rest. He makes a fire and sits next to it. He smokes and chews betelnut. He waits for dinner, once ready he eats with the family and then, he walks to the road junction. Men gather at the road, chew betel nut, smoke and talk. Every two weeks on pay day the bottle shops are full. The bottle shops are enclosed and sell beer through a window. There is no space to sit down and drink. Men usually buy the beer and walk to a convenient space to sit and drink (usually a rock at the side of the road, a bench close to a selling stand or at the beach). Andrew does not stay too long since the later it gets the more drunken men around. He goes back home and sleeps before midnight. On Sunday he wakes up and gets ready for church service. Service starts at 9 am. He and his family have tea in the morning and immediately walk to the church area. After the service they all walk back. Sometimes he stops at the garden and collects betel nut to chew during the week. The family has dinner together at the usual outdoors area. They get visitors on Sundays that come by to share food, betel nut or a smoke. He stays awake until the visitors leave; Andrew drinks tea with them and they talk for hours. They sit outside next to the fire with only the smoke protecting them from mosquito bites. When the visitors leave he goes to sleep usually after midnight. Andrew is not sure how does malaria gets transmitted, he thinks it could be transmitted by a mosquito but other things could also give malaria to a child. In any case malaria is part of life and there is not much he could do about it.

### Supplementary data 10: Adult female (New Ireland Province) – Francisca, 22 years old

Francisca sleeps with her youngest sister and her youngest brother. They share the sleeping area and a LLIN but they do not use it every day. Francisca sleeps wearing a shirt and a laplap. During the week Francisca wakes up at 4 am. Immediately after waking up, she uses the outdoor latrine situated 200m away from the house. Then, she starts the fire and prepares breakfast. She bathes at home with water from the drum. She usually wears a pair of shorts and a t-shirt, her usual work-clothes. Her shoes are flip-flops. Her arms, legs and feet are usually exposed to mosquito bites. She walks 10 minutes to the main road at 5:30 am and waits for the bus or a company car to pass by and take her to work. She works as a loose-fruit-collector at the big oil palm plantation. Her shift starts at 6 am and continues until 2 pm. The plantation blocks are located in a swampy area are treated with insecticide to protect the palms from pests. Once at the plantation she can change the flip-flops to rubber boots if the picking-areas are flooded. After work Francisca gets home at 3 pm. She changes her work clothes to clean ones if she is not going to the garden. She cleans the kitchen and if there is no food for dinner she goes to the garden. Her garden is 15 minutes away. She follows a narrow walking path behind her house to get there. She gets banana or taro and greens. Sometimes she also gets some betel nut to sell outside her house. She has a table next to the road where she sells the betel nut and sometimes home-made doughnuts. Fortnight-Fridays she prepares doughnuts or flour balls to sell. She prepares the dough after work. It needs time to grow before the frying so she let it grow while she goes to the garden. Before cooking dinner with her sisters she fries the dough. Then she sets the table outside the house to start selling as early as possible and until it gets dark. At about 6 pm the family has dinner. They all sit outside nest to the fire. Francisca keeps an eye on the table while she dines. When the selling stand is open she takes turns with her sisters so she can go bathe at the river. She walks towards the river at 7 or 8 pm. She bathes and does her laundry with the help of a flashlight. It takes her about one hour. Right after, she walks back home and attends the selling again. She chats and chews betel nut with people passing by. Francisca never wears repellent, no one in the village does. She closes her little stand at 10 pm or earlier if she sold all the goods. Twice a week, when she is not selling she joins the praying fellowship at 8 or 9 pm. The church area is 20 minutes away from her house. She walks back home at 10 pm and goes to sleep right away.

During the weekend Francisca wakes up at six or seven in the morning. She uses the outdoor latrine and goes to the river. She takes the dirty dishes and cleans them. She goes with her sisters and they also do the laundry. It takes them a little bit longer than one hour. They stay at the river bank with their feet in the water while doing the laundry. If they need to go to the garden they go in the morning after their bath. Some Saturdays they go fishing at sea or they go to the swamp and work the sago. Working the sago takes the whole day at the swamp starting 9 or 10 am till 3 or 4pm. When the tide is low and the swamp is dry they collect kina shells (clams) but they go a few times a year. On fortnight weekend she goes to town (Kavieng) and buys supplies. She wakes up early, walks to the main road and catches the bus at 8 in the morning. She gets to town at 10 am and buys the flour and sugar for the doughnuts among other items. She gets back to the village in the afternoon and rest for a while in the house. She sits on a patch of grass on a mat and chats with her family. Then, she goes to the river and bathes. Francisca and her sisters cook dinner together and the whole family eats and drinks tea while sitting outside next to the fire. On Sundays she wakes up at 7 and she gets ready for church, she usually wears a meri-blouse and laplap to Church. Service starts at 9 am. She goes to the church grounds and stay they until noon. Her family walks back together and gather. She cooks with her sisters and the family eats an early dinner at 4 or 5 pm. After food they all chat and chew betel nut for hours until she gets tired. They spend all this time outside while adding coconut husks to the fire to repel the mosquitoes. Before going to sleep she goes to the well bathes and fetches water for Monday morning. She goes to sleep at 10 or 11pm and she is usually too tired to care about putting up the net and sleeping under it Once a year Francisca helps with the preparations for the Malangan; a big celebration that takes place once a year for three days and three nights. Three months before the festival regular meeting in the afternoon take place in order to arrange all Malangan preparations . Francisca is scared of malaria and thinks mosquitoes are a bad thing. However there are so many the only thing she can do is to light a fire and put lots of coconut husks on it to repel mosquitoes.
